# Odor and Noise Intolerance in Persons with Self-Reported Electromagnetic Hypersensitivity

**DOI:** 10.3390/ijerph110908794

**Published:** 2014-08-27

**Authors:** Steven Nordin, Gregory Neely, David Olsson, Monica Sandström

**Affiliations:** 1 Department of Psychology, Umeå University, SE-901 87 Umeå, Sweden; E-Mail: greg.neely@psy.umu.se; 2 Department of Public Health and Clinical Medicine, Umeå University, SE-901 87 Umeå, Sweden; E-Mails: david.olsson@envmed.umu.se (D.O.); monica.sandstrom@envmed.umu.se (M.S.)

**Keywords:** idiopathic environmental intolerance, environmental illness, chemical sensitivity, noise sensitivity, hyperacusis

## Abstract

Lack of confirmation of symptoms attributed to electromagnetic fields (EMF) and triggered by EMF exposure has highlighted the role of individual factors. Prior observations indicate intolerance to other types of environmental exposures among persons with electromagnetic hypersensitivity (EHS). This study assessed differences in odor and noise intolerance between persons with EHS and healthy controls by use of subscales and global measures of the Chemical Sensitivity Scale (CSS) and the Noise Sensitivity Scale (NSS). The EHS group scored significantly higher than the controls on all CSS and NSS scales. Correlation coefficients between CSS and NSS scores ranged from 0.60 to 0.65 across measures. The findings suggest an association between EHS and odor and noise intolerance, encouraging further investigation of individual factors for understanding EMF-related symptoms.

## 1. Introduction

Symptoms attributed to electromagnetic field (EMF) exposure are regarded as a subtype of idiopathic environmental intolerance (IEI). IEI is a generic term incorporating disorders attributed to the exposure to environmental factors at levels that are below known limits for toxicity, tolerated by the majority of persons, and that are not explained by other somatic or psychiatric conditions [1]. At the WHO workshop in Prague in 2004 the expression IEI-EMF was suggested [2]. The estimated prevalence of EMF-related symptoms ranges from 1.5 to 5%, depending on definition and geographical region [3,4,5,6,7]. Facial skin symptoms are common; as are neurasthenic and cognitive symptoms, but the reports vary considerably between individuals [3,8,9].

Afflicted persons commonly attribute their symptoms to mobile phones (MP), computer monitors, or electrical equipment in general. These intolerances are here referred to as perceived electromagnetic hypersensitivity (EHS). No direct association between EMF exposure and symptoms has been demonstrated. Instead there is support for a nocebo effect in triggering acute symptoms in IEI-EMF [10]. The importance of individual factors has been emphasized, and associations between IEI-EMF and e.g., anxiety, depression, worry and perceived stress have been reported [9,11,12]. High attention to electrical devices with EMF exposure, likely to be triggered by negative affect, such as anxiety, may play an important role by enhancing the nocebo effect. Increased attention to one type of environmental exposure, believed to be hazardous, may result in increased attention also to other types of environmental exposure. It has been suggested that attention in EHS to a large extent is given environmental factors in general, with stress responses resulting in physiological symptoms that are attributed to factors in the environment [13]. It is therefore of interest whether individuals with EHS to a relatively large extent are intolerant also to other environmental factors, for example to odor and noise. In using either two or three questions to assess annoyance to electrical equipment, odorants and sounds, certain individuals have been identified who report having developed intolerances to all three sources [14]. In another study, the prevalence of disturbance from noise from neighbours, ventilation systems and traffic as well as annoyance from car exhausts and tobacco smoke was higher in individuals with EHS compared to referents [3]. Two recent studies provide further indications of a generalized environmental hypersensitivity. From one of the studies a considerable overlap was reported in both self-reported and physician-diagnosed EHS with corresponding intolerances for odor, noise and certain buildings [13]. In the other study, persons with IEI-EMF were found to be similar to persons who scored high when rating their sensitivity to nine other environmental exposures (including odor and noise). The similarities included various aspects of symptomology as well as illness behavior [15]. However, the intolerances in these reviewed studies were assessed with single questions. The indications of a more general environmental intolerance in EHS call for further investigation with thorough quantification of these intolerances.

As with EHS, odor intolerance has been reported to be associated with anxiety, depression, and stress, but also with increased attention to exposure and somatic sensations as well as with modern health worries [16,17,18,19,20,21]. The similarities in symptomology and psychological profile between EMF-related symptoms and odor intolerance are found in both clinical and non-clinical settings [9,11,22,23,24]. Noise intolerance is commonly defined as an “unusual intolerance to ordinary environmental sounds” [25]. As with other types of IEI, the etiology is not fully known, and an association with personality characteristics was suggested early on [26]. Noise intolerance has been reported to be associated with depression, stress, tension, and elevated sensitivity to other sensory stimuli [27,28,29]. Observations suggest that individuals who report noise intolerance also report odor intolerance, which may indicate a general intolerance to external stimuli [30,31,32,33].

The main objective of this study was to compare individuals with self-reported EHS and healthy controls with respect to extent of intolerance to environmental odor/pungency and noise, by use of validated questionnaire instruments. In addition to the olfactory system that mediates odor sensations, the present assessment included intolerance to the sensation of pungency mediated by the chemosomatosensory system. For simplicity we here use the term odor intolerance when referring to both odor and pungency. Confirmation of prior findings of an association between odor and noise intolerance was also investigated. Based on earlier studies showing that anxiety, depression, worry and stress are associated with EHS as well as with intolerance to odor and noise, it was hypothesized that intolerance to odor and noise would be increased in persons with EHS. It was further hypothesized that odor intolerance would correlate with noise intolerance.

## 2. Methods

### 2.1. Participants

Individuals who based on a general question reported symptoms that they associated with use of MP, computer monitors or electrical equipment in general were invited to participate through advertisement in eight Swedish newspapers with potential to reach a large proportion of persons living in Sweden. Those who responded to the advertisement were mailed a questionnaire. Of 160 persons with EMF-related symptoms who responded to the advertisement, 117 agreed to participate. In addition to the general question of symptoms associated with use of MP/wireless phones, computer monitors or electrical equipment, an inclusion criterion was reporting at least one of the specific symptoms in Table 1 when using MP, computer monitors or other electronic equipment. One-hundred and fifteen of the 117 individuals fulfilled this criterion, of which 113 responded to questions about odor and noise intolerance, and thus constituted an EHS group. The prevalence of specific symptoms attributed to MP/wireless phones, computer monitors or other electronic equipment are presented in Table 1. Mean (SD) time of having had EMF-related symptoms in the EHS group was 11.2 (6.7) years.

For each of the 117 person with EMF-attributed symptoms, two controls, matched with respect to age and sex, were recruited through the Swedish population register and sent the same questionnaire. Of these 234 individuals, 106 agreed to participate. Forty-eight of these fulfilled the inclusion criterion of reporting none of the specific symptoms (Table 1) when using MP, computer monitors or other electronic equipment. The EHS and control groups are further described in Table 2. The two groups did not differ significantly in age (*t*-test) or sex distribution (chi^2^-test), but the groups did differ in employment status and work capacity (chi^2^-test; Table 2).

**Table 1:** Number of participants (%) in the group with electromagnetic hypersensitivity who reported specific symptoms attributed to different sources.

Symptom	Mobile/Wireless Phone	Computer Monitors	Other Electronic Equipment
Dizziness	41 (36.3)	25 (22.1)	40 (35.4)
General discomfort	67 (59.3)	45 (39.8)	56 (49.6)
Concentration difficulties	45 (39.8)	41 (36.3)	37 (32.7)
Memory loss	32 (28.3)	24 (21.2)	28 (24.8)
Fatigue	40 (35.4)	43 (38.1)	39 (34.5)
Headache	52 (46.0)	38 (33.6)	36 (31.9)
Warmth behind/around the ear	77 (68.1)	8 (7.1)	13 (11.5)
Warmth on the ear	76 (67.3)	10 (8.8)	10 (8.8)
Burning skin	49 (43.4)	50 (44.2)	42 (37.2)
Tingling/tightness	39 (34.5)	43 (38.1)	36 (31.9)
Sleeping problems	29 (25.7)	28 (24.8)	37 (32.7)
Tinnitus	28 (24.8)	18 (15.9)	25 (22.1)
Numbness	29 (25.7)	29 (25.7)	27 (23.9)

**Table 2:** Self-reported participant characteristics in the groups with electromagnetic hypersensitivity (EHS) and control group.

Characteristic	EHS (n = 113)	Controls (n = 48)	*t*-/chi^2^-Value	*p*-Value
Age, mean (SD)	49.2 (12.5)	51.7 (11.8)	1.16	0.246
Female sex, n (%)	83 (73.5)	35 (72.9)	0.01	0.944
Employment status, n (%)	43 (38.1)	32 (66.7)	17.96	<0.001
Full or part time employment				
Full or part time sick leave	25 (22.1)	3 (6.2)		
Disability pension	30 (26.5)	3 (6.2)		
Unemployed or retired	15 (13.3)	7 (14.6)		
Not responding	0 (0)	3 (6.2)		
Work capability, n (%)			8.20	0.017
Good	51 (58.6)	33 (84.6)		
Moderate	17 (19.5)	3 (7.7)		
Moderate	19 (21.8)	3 (7.7)		

### 2.2. Questionnaire Instruments

The questionnaire included questions about specific symptoms attributed to EMF sources (Table 1), demographics, employment status and work capacity (Table 2), time of having had EMF-related symptoms, and intolerance to odor and noise. Odor intolerance was assessed with the Chemical Sensitivity Scale (CSS) [32], and noise intolerance with the Noise Sensitivity Scale (NSS) [26]. The CSS and NSS are analogous tools for quantifying self-reported affective reactions and behavioral disruptions in daily activities caused by odorous/pungent environmental substances and by noise, respectively. They consist of 21 statements each (e.g., “At movies, other persons’ perfume and aftershave disturb me” and “At movies, whispering and crinkling candy wrappers disturb me”) to be responded to on a six-point Likert scale and summed to give an overall score that ranges from 1 to 105 (high score indicating high sensitivity). Both the CSS and NSS have good test-retest reliability, internal consistency, and predictive validity, and generate approximately normal distributions [26,32,34,35]. The CSS has two subscales for each of its two dimensions [32]. The dimension symptom type consists of the subscales sensory/somatic symptoms (13 items; e.g., “I would not mind living in an apartment that has a weak smell”) and neurasthenic symptoms (8 items; e.g., “Even food odors that I normally like will bother me if I am trying to concentrate”), and the dimension reactivity consists of the subscales negative affective reactions (12 items; e.g., “I am easily alerted by odorous/pungent substances”) and behavioral disruptions (8 items; e.g., “If it is smelly/pungent where I am studying, I try to shut it out or move someplace else”). The last item is of general character (“I am sensitive to odorous/pungent substances”). A global measure incorporates all 21 items. Each of the 21 item statements about the noise environment in the NSS have their corresponding statement about the odor environment in the CSS, being as similar as possible, and the response alternatives in the two scales are identical for each corresponding statement [32]. It was therefore assumed that the item statements in the NSS would constitute the same four subscales of the CSS, but referring to noise environments. The internal consistency in the present study (n = 161) for the CSS and NSS, respectively, was 0.89 and 0.88 for the subscale sensory symptoms, 0.86 and 0.83 for neurasthenic symptoms, 0.87 and 0.81 for affective reactions, 0.88 and 0.89 for behavioral disruptions, and 0.94 and 0.92 for the global measures.

### 2.3. Procedure

Nonresponders in both groups received one reminder. The data collection was carried out during a period of five months (December 2005–April 2006). Informed consent was obtained from each participant, and they were paid for their participation. Ethical approval was given by the Regional Ethical Research Board at Umeå University.

### 2.4. Statistical Analysis

The EHS and control groups were compared on the variables that describe participant characteristics (Table 2) using *t*-test and chi-square test, and on the CSS and NSS subscales and global measures using *t*-test (α = 0.05). Pearson correlation coefficients were calculated between scores on the CSS and NSS for the subscales and global measures for the two participant groups separately and for all participants (α = 0.01). All variables were approximately normally distributed. The statistical analyses were performed using SPSS Statistics 21 for Windows (IBM Corporation, Armonk, NY, USA).

## 3. Results

Mean scores on the CSS and NSS for each participant group are shown in Table 3. Results from the *t*-tests (Table 2) show that the EHS group had significantly higher scores than the controls on the five CSS and five NSS scales. Pearson correlation coefficients between scores on the CSS and NSS for the subscales and global measures for the two participant groups separately and for all participants are given in Table 4. For the two groups the coefficients ranged between 0.54 and 0.66, and for all participants between 0.60 and 0.65. All correlations were statistically significant at *p* < 0.001.

**Table 3:** Mean (SD) scores on the subscales and global measures of the Chemical Sensitivity Scale (CSS) and Noise Sensitivity Scale (NSS) in the group with electromagnetic hypersensitivity (EHS) and the control group. Results from *t*-tests comparing the two groups are given.

Sensitivity Scale	EHS (n = 113)	Controls (n = 48)	*t*-Value	*p*-Value
**CSS**				
Symptom				
Sensory/somatic	32.8 (10.0)	29.5 (8.5)	2.07	0.041
Neurasthenic	12.5 (5.4)	10.7 (4.1)	2.31	0.023
Reactivity				
Affective	39.0 (11.3)	35.4 (9.6)	2.07	0.041
Behavioral	21.5 (8.5)	18.4 (7.3)	2.30	0.023
Global	63.9 (20.3)	56.7 (16.7)	2.34	0.021
**NSS**				
Symptom				
Sensory/somatic	34.2 (10.0)	27.5 (8.9)	4.26	<0.001
Neurasthenic	15.6 (5.3)	12.4 (4.6)	3.84	<0.001
Reactivity				
Affective	35.4 (10.0)	29.4 (7.9)	4.04	<0.001
Behavioral	24.7 (8.4)	19.8 (7.4)	3.68	<0.001
Global	63.4 (18.8)	51.6 (15.4)	4.15	<0.001

**Table 4:** Pearson correlation coefficients between scores on the subscales and global measure of the Chemical Sensitivity Scale and the Noise Sensitivity Scale for the group with electromagnetic hypersensitivity (EHS), the control group, and the overall group.

Sensitivity Scale	EHS (n = 113)	Controls (n = 48)	Overall (n = 161)
Symptom			
Sensory/somatic	0.61	0.62	0.63
Neurasthenic	0.66	0.59	0.65
Reactivity			
Affective	0.59	0.57	0.60
Behavioral	0.63	0.54	0.62
Global	0.63	0.61	0.64

All correlations were significant at *p* < 0.001.

## 4. Discussion

Prior research, in which environmental intolerances were assessed using only single questions, indicate that individuals with symptoms attributed to EMF may also suffer from complaints related to environmental odor and noise [3,13,14,15]. This motivated the present study using valid and reliable questionnaire instruments for quantifying odor and noise intolerance.

In accordance with the hypotheses, perceived intolerance to odor/pungency and noise was found to be more common among persons with EHS compared to healthy controls. The results correspond with previous observations of hyperresponsiveness to sensory stimuli in subjects with EHS [36,37,38]. Deviations in heart rate variability, suggesting autonomic nervous system (ANS) changes toward elevation in sympathetic tone, have been found in EHS without MP-related symptoms during rest as well as in response to induced stress; whereas a similar investigation of subjects with MP-related symptoms revealed signs of elevation in sympathetic tone only during induced stress [37,38,39].

It is of interest to note that the effects sizes, expressed as *t*-values, were larger for the NSS scales than for the CSS scales, suggesting that intolerance in EHS is higher for noise than for odor. A link between noise intolerance and EMF-related symptoms may pertain to tinnitus. This condition is common among patients with noise intolerance, and has been shown to be relatively common also in EMF-related symptoms [40,41]. It has been suggested that vulnerability, probably due to an over-activated cortical distress network, may be responsible for both tinnitus and EMF-related symptoms [41]. In the present study group, the prevalence of self-reported tinnitus (not restricted to use of electrical equipment) at least on a weekly basis was as high as 35% in the EHS group, to be compared with none of the participants in the control group. However, these speculations call for further investigation.

The data suggest that persons with EHS are high on odor and noise intolerance in a broad sense, since the EHS group scored higher than the controls on all four subscales of the CSS and NSS. Thus, the intolerance to odor/pungency and noise included the subscales sensory symptoms such as annoyance and disturbance, neurasthenic symptoms such as concentration difficulties and nervousness, affective reactions such as becoming alerted and irritated, and behavioral disruptions such as shutting out the exposure and moving someplace else. Normative data is available for the CSS (but not for the NSS), which has a mean (SD) of 62.3 (15.2) [42]. As would be expected for the normative data that is based on a population sample in which individuals with odor intolerance are not excluded, it is lower than that for the EHS group and higher than that for the control group.

The overlap between self-reported EMF-related symptoms and odor and noise intolerance favors the notion that individual factors play an important role in EMF-related symptoms. Such factors may include anxiety, depression and stress that have previously been reported in this particular group of individuals, but also ANS derangement and general hyperresponsiveness to sensory stimuli. However, importantly, the cross-sectional nature of the data does not enable conclusions about cause and effect. It cannot be excluded that anxiety, depression, stress and exhaustion, as well as physiological changes may be consequences of living with EMF-related symptoms. A second purpose of the study was to verify prior findings of an association between odor and noise intolerance. Fairly strong correlations (r = 0.60–0.65) for the five measures of intolerance based on all participants can be considered to verify earlier observations [30,32].

Data from a Swedish population-based study show that 58% of those with self-reported EHS also experienced hypersensitivity to odors, noise and/or certain buildings [13]. Results from that study, as well as other studies [3,4,14,15,43], suggest large overlap in prevalence between environmental hypersensitivities in general. Interestingly, there is also large overlap in prevalence between environmental hypersensitivity and other forms of medically unexplained conditions for which ANS imbalance has been proposed as contributing to the clinical picture, such as fibromyalgia, chronic fatigue syndrome and irritable bowel syndrome [44,45,46]. Sensitization has been proposed as an explanatory model for many of these forms of medically unexplained symptoms [47]. Sensitization, the increase in response to repeated exposure, is the opposite of habituation and may manifest at a cellular, systemic or cognitive level. Cognitive sensitization refers to an increased attention to symptoms and symptom-related environmental threats. At all levels sensitization may increase somatic sensations and cause or aggravate pre-existing complaints [48,49]. Sensitization has also been suggested to activate the hypothalamic-pituitary-adrenal (HPA) axis and shift the ANS activity in a direction toward hypersympathotone, explaining many of the symptoms in the medically unexplained conditions such as those mentioned [50]. Support for effects on the HPA axis in EHS has been provided by elevated levels of arousal in EHS [51], and for effects on the ANS in terms of a general imbalance in its regulation in EHS [37,38,39,52]. The tight link between emotional factors (e.g., anxiety and stress), HPA axis activation, and increased sympathetic nervous system activity may explain many of the symptoms commonly reported by individuals with EMF-related symptoms (e.g., general discomfort, difficulties concentrating, memory loss, heart palpitation, paresthesia, sleeping disorders). The concept of cognitive sensitization is of interest concerning the differences in perceived intolerance to environmental factors other than EMF, such as odor and noise, and when addressing the question why EMF-related symptoms generalize in some individuals but not in others. The possibility of sensitization (based predominantly on visual and auditory cues of electrical equipment) in EHS does not exclude the possibility of a nocebo effect, and that high attention to electrical devices with potential EMF exposure may play an important role by enhancing sensitization as well as a nocebo effect. This would imply that attention in EHS to a large extent may be given environmental factors in general.

Limited representativeness in the present study calls for caution. Thus, only 46% of the invited participants from the population register agreed to participate, and the sample of EHS cases was not population-based. Rather than representing EHS cases in general, the EHS sample is likely to have had fairly severe degree of hypersensitivity attributed to EMF since the symptom prevalence, varying depending on type of symptom, in general was quite high. The prevalence ranged between 25 and 68% for MP/wireless phones, between 7 and 44% for computer monitors, and between 9 and 50% for other electric equipment (Table 1). The high symptom prevalence together with the EHS group having had EMF-related symptoms for quite a long time (on average about 11 years) may explain the relatively large proportion of the EHS cases reporting sick leave (22%) and disability pension (26%).

An implication of the present findings is that individual factors appear to play an important role in EHS/IEI-EMF. The results support the notion that different types of environmental intolerance share similar underlying mechanisms, which may possibly include a nocebo effect and sensitization, although further studies are needed to clarify these issues.

## 5. Conclusions

The results suggest that symptoms attributed to EMF are associated with odor/pungency and noise intolerance. Furthermore, prior findings of an association between odor and noise intolerance was verified. Taken together, the findings encourage further investigation of individual factors for understanding EMF-related symptoms as well as other types of medically unexplained symptoms.

## References

[1] IPCS/WHO (1996). Conclusions and recommendations of a workshop on multiple chemical sensitivities (MCS). Regulat. Toxicol. Pharmacol..

[2] Hansson Mild K., Repacholi M., van Deventer E., Ravazzani P. (2004). WHO International Workshop on Electromagnetic Field Hypersensitivity.

[3] Hillert L., Berglind N., Arnetz B.B., Bellander T. (2002). Prevalence of self-reported hypersensitivity to electric or magnetic fields in a population-based questionnaire survey. Scand. J. Work. Environ. Health..

[4] Levallois P., Neutra R., Lee G., Hristova L. (2002). Study of self-reported hypersensitivity to electromagnetic fields in California. Environ. Health. Perspect..

[5] Schreier N., Huss A., Roosli M. (2006). The prevalence of symptoms attributed to electromagnetic field exposure: A cross-sectional representative survey in Switzerland. Soz. Praventivmed..

[6] Eltiti S., Wallace D., Zougkou K., Russo R., Joseph S., Rasor P., Fox E. (2007). Development and evaluation of the electromagnetic hypersensitivity questionnaire. Bioelectromagnetics.

[7] Schröttner J., Leitgeb N. (2008). Sensitivity to electricity: Temporal changes in Austria. BMC Public Health.

[8] Seitz H., Stinner D., Eikmann T., Herr C., Roosli M. (2005). Electromagnetic hypersensitivity (EHS) and subjective health complaints associated with electromagnetic fields of mobile phone communication: A literature review published between 2000 and 2004. Sci. Total. Environ..

[9] Johansson A., Nordin S., Heiden M., Sandström M. (2005). Symptoms, personality traits, and stress in people with mobile phone-related symptoms and electromagnetic hypersensitivity. J. Psychosom. Res..

[10] Rubin G.J., Nieto-Hernandez R., Wessely S. (2010). Idiopathic environmental intolerance attributed to electromagnetic fields (formerly ‘electromagnetic hypersensitivity’): An updated systematic review of provocation studies. Bioelectromagnetics.

[11] Bergdahl J. (1995). Psychologic aspects of patients with symptoms presumed to be caused by electricity or visual display units. Acta. Odontol. Scand..

[12] Bergdahl J., Marell L., Bergdahl M., Perris H. (2005). Psychobiological personality dimensions in two environmental-illness patient groups. Clin. Oral. Invest..

[13] Palmquist P., Claeson A.-S., Neely G., Stenberg B., Nordin S. (2014). Overlap in prevalence between various types of environmental intolerance. Int. J. Hyg. Environ. Health.

[14] Carlsson Eek F. (2005). Subjective Annoyance Attributed to Electricity and Smells: Epidemiology and Stress Physiology. Ph.D. Thesis.

[15] Baliatsas C., Van Kamp I., Hooiveld M., Yzermans J., Lebret E. (2014). Comparing non-specific physical symptoms in environmentally sensitive patients: Prevalence, duration, functional status and illness behavior. J Psychosom Res..

[16] Sparks P.J., Daniell W., Black D.W., Kipen H.M., Altman L.C., Simon G.E., Terr A.I. (1994). Multiple chemical sensitivity syndrome: A clinical perspective. I. Case definition, theories of pathogenesis, and research needs. J. Occup. Med..

[17] Black D.W. (2000). The relationship of mental disorders and idiopathic environmental intolerance. Occup. Med..

[18] Bailer J., Witthoft M., Rist F. (2008). Modern health worries and idiopathic environmental intolerance. J. Psychosom. Res..

[19] Bailer J., Witthoft M., Rist F. (2008). Psychological predictors of short- and medium term outcome in individuals with idiopathic environmental intolerance (IEI) and individuals with somatoform disorders. J. Toxicol. Environ. Health A.

[20] Andersson L., Bende M., Millqvist E., Nordin S. (2009). Attention bias and sensitization in chemical sensitivity. J. Psychosom. Res..

[21] Skovbjerg S., Zachariae R., Rasmussen A., Johansen J.D., Elberling J. (2009). Attention to bodily sensations and symptom perception in individuals with idiopathic environmental intolerance. Environ. Health Prev. Med..

[22] Bergdahl J., Anneroth G., Stenman E. (1994). Description of persons with symptoms presumed to be caused by electricity or visual display units: Oral aspects. Scand. J. Dental. Res..

[23] sterberg K., Persson R., Karlson B., Carlsson Eek F., Orbaek P. (2007). Personality, mental distress, and subjective health complaints among persons with environmental annoyance. Human Exp. Toxicol..

[24] Persson R., Carlsson Eek F., Osterberg K., Orbaek P., Karlson B. (2008). A two-week monitoring of self-reported arousal, worry and attribution among persons with annoyance attributed to electrical equipment and smells. Scand. J. Psychol..

[25] Vernon J.A. (1987). Pathophysiology of tinnitus: A special case: Hyperacusis and a proposed treatment. Am. J. Otol..

[26] Weinstein N.D. (1978). Individual differences in reactions to noise: A longitudinal study in a college dormitory. J. Appl. Psychol..

[27] Stansfeld S.A., Clark C.R., Jenkins L.M., Tarnopolsky A. (1985). Sensitivity to noise in a community sample: I. Measurement of psychiatric disorder and personality. Psychol. Med..

[28] Marriage J., Barnes N.M. (1995). Is central hyperacusis a symptom of 5-hydroxytryptamine (5-HT) dysfunction?. J. Laryngol. Otol..

[29] Andersson G., Lindvall N., Hursti T., Carlbring P. (2002). Hypersensitivity to sound (hyperacusis): A prevalence study conducted via the Internet and post. Int. J. Audiol..

[30] Bell I.R., Hardin E.E., Baldwin C.M., Schwartz G.E. (1995). Increased limbic system symptomatology and sensitizability of young adults with chemical and noise sensitivities. Environ. Res..

[31] Szarek M.J., Bell I.R., Schwartz G.E. (1997). Validation of a brief screening measure of environmental chemical sensitivity: The chemical odor intolerance index. J. Environ. Psychol..

[32] Nordin S., Millqvist E., Löwhagen O., Bende M. (2003). The Chemical Sensitivity Scale: Psychometric properties and comparison with the noise sensitivity scale. J. Environ. Psychol..

[33] Nordin S., Körning Ljungberg J., Claeson A.-S., Neely G. (2013). Stress and odor sensitivity in persons with noise sensitivity. Noise Health..

[34] Topf M. (1985). Personal and environmental predictors of patient disturbance due to hospital noise. J. Appl. Psychol..

[35] Ekehammar B., Dornic S. (1990). Weinstein’s Noise Sensitivity Scale: Reliability and construct validity. Percept. Mot. Skills..

[36] Sandström M., Lyskov E., Berglund A., Medvedev S., Hansson Mild K. (1997). Neurophysiological effects of flickering light in patients with perceived electrical hypersensitivity. J. Occup. Environ. Med..

[37] Lyskov E., Sandström M., Hansson Mild K. (2001). Neurophysiological study of patients with perceived ‘electrical hypersensitivity’. Int. J. Psychophysiol..

[38] Lyskov E., Sandström M., Hansson Mild K. (2001). Provocation study of persons with perceived electrical hypersensitivity and controls using magnetic field exposure and recording of electrophysiological characteristics. Bioelectromagnetics.

[39] Wilén J., Johansson A., Kalezic N., Lyskov E., Sandström M. (2006). Psychophysiological tests and provocation of subjects with mobile phone related symptoms. Bioelectromagnetics.

[40] Nelson J.J., Chen K. (2004). The relationship of tinnitus, hyperacusis, and hearing loss. Ear Nose Throat J..

[41] Landgrebe M., Frick U., Hauser S., Hajak G., Langguth B. (2009). Association of tinnitus and electromagnetic hypersensitivity: Hints for a shared pathophysiology?. PLoS One.

[42] Nordin S., Bende M., Millqvist E. (2004). Normative data of the Chemical Sensitivity Scale. J. Environ. Psychol..

[43] Eriksson N.M., Stenberg B.G. (2006). Baseline prevalence of symptoms related to indoor environment. Scand. J. Public Health.

[44] Barsky A.J., Borus J.F. (1999). Functional somatic syndromes. Ann. Intern. Med..

[45] Wessely S., Nimuan C., Sharpe M. (1999). Functional somatic syndromes: One or many?. Lancet.

[46] Jason L.A., Taylor R.R., Kennedy C.L. (2000). Chronic fatigue syndrome, fibromyalgia, and multiple chemical sensitivities in a community-based sample of persons with chronic fatigue syndrome-like symptoms. Psychosom. Med..

[47] Eriksen H.R., Ursin H. (2004). Subjective health complaints, sensitization, and sustained cognitive activation (stress). J. Psychosom. Res..

[48] Brosschot J.F. (2002). Cognitive-emotional sensitization and somatic health complaints. Scand. J. Psychol..

[49] Verkuil B., Brosschot J.F., Thayer J.F. (2007). A sensitive body or a sensitive mind? Associations among somatic sensitization, cognitive sensitization, health worry, and subjective health complaints. J. Psychosom. Res..

[50] Deary V., Chalder T., Sharpe M. (2007). The cognitive behavioural model of medically unexplained symptoms: A theoretical and empirical review. Clin. Psychol. Rev..

[51] Eltiti S., Wallace D., Ridgewell A., Zougkou K., Russo R., Sepulveda F., Mirshekar-Syahkal D., Rasor P., Deeble R., Fox E. (2007). Does short-term exposure to mobile phone base station signals increase symptoms in individuals who report sensitivity to electromagnetic fields? A double-blind randomized provocation study. Environ. Health Perspect..

[52] Levitt B.B., Lai H. (2010). Biological effects from exposure to electromagnetic radiation emitted by cell tower base stations and other antenna arrays. Environ Rev..

